# *In vitro* activity of SPR206 and comparator agents against *Pseudomonas aeruginosa*, *Acinetobacter* species, and Enterobacterales responsible for infection in United States, European, Israeli, and Turkish hospitals

**DOI:** 10.1128/spectrum.01341-25

**Published:** 2025-09-10

**Authors:** Gina M. Morgan, Ian Critchley, Nicole Cotroneo, Helio Sader, Mariana Castanheira, Rodrigo Mendes

**Affiliations:** 1 JMI Laboratories/Element Materials Technology 138461, North Liberty, Iowa, USA; 2 Spero Therapeutics 612305, Cambridge, Massachusetts, USA; University of Manitoba, Winnipeg, Manitoba, Canada

**Keywords:** SPR206, carbapenem resistance, polymyxin, CRE, multidrug-resistant, extensively drug-resistant, DTR

## Abstract

Increasing rates of antimicrobial resistance require additional safe and effective options for managing difficult-to-treat infections. SPR206 is a next-generation polymyxin with improved *in vivo* safety profiles. This study determined the *in vitro* activity of SPR206 against a diverse collection of gram-negative isolates. SPR206 was tested against 5,179 Enterobacterales, *Pseudomonas aeruginosa*, and *Acinetobacter* spp. collected from patients in the United States (US), Europe, Israel, and Turkey. Broth microdilution was performed following the Clinical & Laboratory Standards Institute (CLSI) guidelines, and MIC results interpreted by CLSI, European Committee on Antimicrobial Susceptibility Testing (EUCAST), and United States Food and Drug Administration (US FDA) criteria. SPR206 (MIC_50/90_, 0.25/0.25–0.5 mg/L) was active against *P. aeruginosa*, including the multidrug-resistant, extensively drug-resistant, and difficult-to-treat subsets, with 99.8–100% of isolates inhibited at MIC values of ≤2 mg/L. SPR206 inhibited 94.5% of *Acinetobacter* spp., including 90.4 and 87.9% of the multidrug-resistant (MIC_50/90_, 0.12/2 mg/L) and extensively drug-resistant (MIC_50/90_, 0.12/8 mg/L) subsets at MIC values of ≤2 mg/L. SPR206 (MIC_50/90_, 0.06/0.25 mg/L) inhibited 95.4% of non-Morganellaceae Enterobacterales at MIC values of ≤2 mg/L. SPR206 MIC_50_ and MIC_90_ values of 0.06 and 0.5 mg/L were obtained against carbapenem-resistant Enterobacterales from the US and Western Europe, whereas MIC_50_ of 0.5 mg/L and MIC_90_ of 64 mg/L were noted against carbapenem-resistant Enterobacterales isolates from Eastern Europe. SPR206 demonstrated potent activity against a large collection of gram-negative organisms, including resistant subsets. These results, along with an enhanced safety profile, warrant the development of SPR206 as a candidate for the treatment of infections caused by gram-negative bacteria.

**IMPORTANCE:**

SPR206 is a novel, next-generation polymyxin with improved safety profiles that demonstrates activity against gram-negative organisms. This study benchmarks the activity and spectrum of SPR206 against a large collection of gram-negative isolates, including multidrug-resistant and extensively drug-resistant *Acinetobacter* spp. and *Pseudomonas aeruginosa*, and carbapenem-resistant Enterobacterales collected from multiple medical sites in the US and Europe. Additional treatment options are needed as antimicrobial resistance emerges and spreads. The results presented in this manuscript show potent SPR206 activity against resistant and difficult-to-treat gram-negative organisms.

## INTRODUCTION

Increasing rates of antimicrobial resistance are occurring worldwide and becoming an urgent public health concern. In 2019 alone, infections associated with antimicrobial-resistant pathogens were estimated to have caused approximately 1.3 million deaths internationally (1–3). The frequency of bacteria developing antimicrobial resistance has increased, likely driven by extensive, prolonged use, as well as misuse of antimicrobial agents, and hindered the efficacy of various agents (4, 5). Cross-resistance and co-resistance across antimicrobial classes have also been observed (6, 7), along with multidrug resistance (MDR) phenotypes, and become increasingly common in gram-negative organisms, such as *Escherichia coli*, *Klebsiella* spp., *Pseudomonas aeruginosa*, and *Acinetobacter baumannii-calcoaceticus* complex (8, 9), causing difficult-to-treat resistance infections (DTR). The United States Centers for Disease Control and Prevention (US CDC) classifies MDR *P. aeruginosa* as a serious threat, whereas carbapenem-resistant Enterobacterales (CRE) and carbapenem-resistant *A. baumannii* are considered urgent threats that challenge healthcare and life expectancy (3).

Polymyxins, such as colistin and polymyxin B, remain as options for treating many infections caused by gram-negative MDR bacteria. However, these drugs are particularly nephrotoxic and can cause neurotoxicity and hypersensitivity, and dose optimization at an individual patient level is essential (10). SPR206 is a novel intravenous next-generation polymyxin derivative for treating hospital-acquired or ventilator-associated bacterial pneumonia (HABP/VABP) caused by CRE, carbapenem-resistant *A. baumannii-calcoaceticus* complex, and carbapenem-resistant *P. aeruginosa* (11, 12). Different from other polymyxins due to molecule structural changes, SPR206 showed potent *in vitro* activity against MDR Enterobacterales, CRE, and MDR *A. baumannii* and *P. aeruginosa* (13–15), as well as reduced bacterial burden in an *in vivo* mouse model (16). In clinical trials, SPR206 demonstrated reduced renal cytotoxicity and was relatively safe and well-tolerated by patients, including those with moderate and severe renal impairment (12, 17*).*

This study determined the *in vitro* activity of SPR206 and comparator agents against a diverse and representative group of surveillance Enterobacterales, *P. aeruginosa*, and *Acinetobacter* spp. isolates causing infections in patients hospitalized in medical centers in the United States, Europe, Israel, and Turkey during 2021.

## MATERIALS AND METHODS

### Organisms tested

A total of 5,179 isolates recovered from documented infections in 2021 were included in this study, and the species are described in Table 1. These isolates were part of the SENTRY Antimicrobial Surveillance Program for 2021 and collected in 30 medical centers (2,541 isolates; 49.1% overall) located in the nine Census Divisions in the USA and 35 medical centers in Europe, Israel, and Turkey (2,638 isolates; 50.9% overall). The collected isolates were primarily responsible for pneumonia in hospitalized patients (1,828 isolates; 35.3% overall), bloodstream infection (1,209 isolates; 23.4% overall), urinary tract infections (995 isolates; 19.2% overall), skin and skin structure infections (769 isolates; 14.9%), and intra-abdominal infections (281 isolates; 5.4% overall) (18). Bacterial species were identified by the participating clinical laboratories and confirmed by Element Iowa City (JMI Laboratories) using standard microbiology methods and matrix-assisted laser desorption ionization-time of flight mass spectrometry (Bruker Daltonics, Bremen, Germany).

**TABLE 1:** MIC and cumulative percent distribution of SPR206^*f*^^,^^*g*^

Organism/organism group (no. of isolates)	No. and cumulative % of isolates inhibited at MIC (mg/L) of:													MIC_50_	MIC_90_
	≤0.03	0.06	0.12	0.25	0.5	1	2	4	8	16	32	64	>64		
*Pseudomonas aeruginosa* (898)	5 0.6	31 4.0	280 35.2	530 94.2	43 99.0	5 99.6	2 99.8	0 99.8	2 100					0.25	0.25
Carbapenem-resistant (141)	1 0.7	8 6.4	29 27.0	87 88.7	14 98.6	2 100								0.25	0.5
DTR (34)	0 0.0	2 5.9	7 26.5	16 73.5	8 97.1	1 100								0.25	0.5
MDR (150)	0 0.0	12 8.0	32 29.3	83 84.7	21 98.7	2 100								0.25	0.5
XDR (75)	0 0.0	5 6.7	18 30.7	35 77.3	16 98.7	1 100								0.25	0.5
*Acinetobacter* spp.^*a*^ (724)	40 5.5	242 39.0	212 68.2	111 83.6	50 90.5	14 92.4	15 94.5	10 95.9	2 96.1	11 97.7	7 98.6	4 99.2	6 100	0.12	0.5
Carbapenem-resistant (332)	12 3.6	142 46.4	80 70.5	34 80.7	17 85.8	8 88.3	5 89.8	6 91.6	1 91.9	10 94.9	7 97.0	4 98.2	6 100	0.12	4
Colistin-resistant (54)	0 0.0	0 0.0	5 9.3	5 18.5	2 22.2	2 25.9	4 33.3	6 44.4	2 48.1	11 68.5	7 81.5	4 88.9	6 100	16	>64
MDR (353)	12 3.4	148 45.3	87 70.0	37 80.5	21 86.4	9 89.0	5 90.4	6 92.1	1 92.4	10 95.2	7 97.2	4 98.3	6 100	0.12	2
XDR (273)	8 2.9	122 47.6	64 71.1	25 80.2	15 85.7	3 86.8	3 87.9	5 89.7	1 90.1	10 93.8	7 96.3	4 97.8	6 100	0.12	8
Enterobacterales^*b*^ (3,228)	509 15.8	1,382 58.6	529 75.0	134 79.1	37 80.3	11 80.6	6 80.8	5 80.9	11 81.3	26 82.1	23 82.8	21 83.5	534 100	0.06	64
Excluding resistant spp^*c*^ (2,734)	509 18.6	1,382 69.2	528 88.5	134 93.4	37 94.7	11 95.1	6 95.4	5 95.5	8 95.8	18 96.5	18 97.1	14 97.7	64 100	0.06	0.25
* Escherichia coli* (850)	199 23.4	525 85.2	110 98.1	13 99.6	0 99.6	0 99.6	2 99.9	1 100						0.06	0.12
* Klebsiella pneumoniae* (850)	93 10.9	410 59.2	246 88.1	45 93.4	9 94.5	5 95.1	1 95.2	1 95.3	4 95.8	15 97.5	10 98.7	5 99.3	6 100	0.06	0.25
Colistin-resistant^*d*^ (131)	0 0.0	2 1.5	2 3.1	1 3.8	0 3.8	1 4.6	0 4.6	4 7.6	8 13.7	18 27.5	17 40.5	14 51.1	64 100	64	>64
CRE^*e*^ (91)	7 7.7	41 52.7	9 62.6	8 71.4	4 75.8	2 78.0	0 78.0	0 78.0	1 79.1	7 86.8	6 93.4	3 96.7	3 100	0.06	32
MDR (331)	38 11.5	155 58.3	55 74.9	17 80.1	6 81.9	2 82.5	1 82.8	2 83.4	3 84.3	10 87.3	7 89.4	6 91.2	29 100	0.06	64

^*a*^ Includes *A. baumannii-calcoaceticus* species complex (627), *A. beijerinckii* (1), *A. berezinae* (14), *A. courvalinii* (4), *A. gerneri* (1), *A. guillouiae* (1), *A. gyllenbergii* (1), *A. haemolyticus* (1), *A. johnsonii* (8), *A. junii* (10), *A. lwoffii* (7), *A. proteolyticus* (1), *A. radioresistens* (12), *A. schindleri* (1), *A. soli* (2), *A. ursingii* (24), *A. variabilis* (1), *A. vivianii* (1), and *Acinetobacter* spp (7).

^*b*^ Include *Citrobacter amalonaticus/farmeri* (1), *Citrobacter freundii species complex* (120), *C. koseri* (120), *Cronobacter sakazakii* (1), *Enterobacter cloacae* species complex (428), *Escherichia coli* (850), *Hafnia alvei* (3), *Klebsiella aerogenes* (130), *K. oxytoca* (220), *K. pneumoniae* (850), *K. variicola* (6), *Kluyvera ascorbata* (1), *Morganella morganii* (120), *Pantoea agglomerans* (1), *Pluralibacter gergoviae* (1), *Proteus mirabilis* (120), *P. penneri* (1), *P. vulgaris* (3), *P. vulgaris* group (7), *Providencia rettgeri* (12), *P. stuartii* (18), *Raoultella ornithinolytica* (2), *Serratia liquefaciens* (1), *S. liquefaciens complex* (1), *S. marcescens* (210), and *S. odorifera* (1).

^*c*^ Excludes species intrinsically resistant to polymyxins (i.e., *Morganella* spp., *Providencia* spp., *Proteus* spp., and *Serratia* spp.).

^*d*^ Enterobacterales isolates resistant to colistin, excluding those species intrinsically resistant to polymyxins.

^*e*^ Includes *Citrobacter freundii* species complex (1), *Enterobacter cloacae* species complex (8), *Escherichia coli* (1), *Klebsiella aerogenes* (5), *K. oxytoca* (3), *K. pneumoniae* (71), and *Serratia marcescens* (2).

^*f*^ Abbreviations: DTR, difficult-to-treat resistant; MDR, multidrug-resistant; XDR, extensively drug-resistant; CRE, carbapenem-resistant Enterobacterales.

^*g*^ Empty cells indicate that the organism group was inhibited at a lower SPR206 MIC.

### Antimicrobial susceptibility testing

Minimum inhibitory concentrations (MICs) against selected isolates were determined by reference broth microdilution (BMD) method, as described by Clinical and Laboratory Standards Institute (CLSI) M07 (19). BMD was performed in cation-adjusted Mueller-Hinton broth (CAMHB) for SPR206 and comparator agents. BMD MIC panels containing all tested agents were manufactured using the Agilent Bravos robot. Panels containing doubling dilution ranges for SPR206 and colistin were produced in a similar manner. Stock solutions of SPR206 and colistin were prepared in concentrations of 5,120 and 1,280 mg/L, respectively, in Falcon conical tubes using water as solvent. Testing concentrations were prepared by diluting the working intermediate solutions also in water using the Agilent Bravos robot and ultra-low retention pipette tips (Labcon; catalog # 1065-960-008-9) in standard untreated polystyrene 96-well plates (Sarstedt catalog #82.1582). The CLSI and European Committee on Antimicrobial Susceptibility Testing (EUCAST) interpretive criteria were applied according to current guidelines (20, 21), and the US Food and Drug Administration (FDA) criteria were applied to tigecycline (22). Quality control (QC) American Type Culture Collection (ATCC) strains were tested concurrently, and acceptable MIC ranges were those published by the CLSI (20).

### Resistance phenotype definitions

CRE was defined as any Enterobacterales displaying imipenem and/or meropenem MIC values at ≥4 mg/L (20). Only meropenem was used to categorize members of the Morganellaceae family due to their intrinsic, decreased susceptibility to imipenem. Isolates showing nonsusceptible phenotypes to at least one agent belonging to three or more drug classes of antibiotics were designated as MDR, and those showing nonsusceptibility to at least one agent in all, but two, drug classes were assigned as extensively drug-resistant (XDR) (23). In addition, *P. aeruginosa* nonsusceptible to piperacillin-tazobactam, ceftazidime, cefepime, aztreonam, meropenem, imipenem-cilastatin, ciprofloxacin, and levofloxacin was considered as DTR isolates (24).

## RESULTS

### Pseudomonas aeruginosa

**Fig 1:**
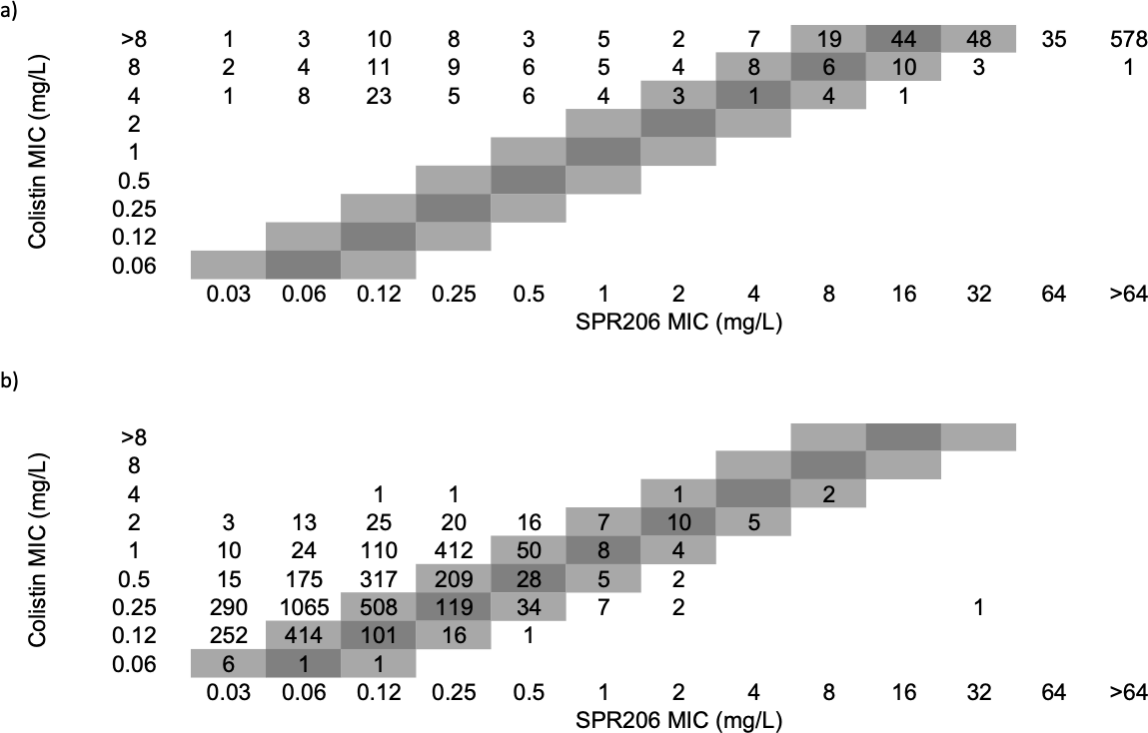
Scatterplot of SPR206 versus colistin MIC (mg/L) results against (a) colistin-resistant and (b) colistin-susceptible *P. aeruginosa, Acinetobacter* spp., and Enterobacterales. *P. aeruginosa* resistant to colistin was not detected.

SPR206 MIC_50_ and MIC_90_ values of 0.25 mg/L were obtained against *P. aeruginosa*, and 99.8% of isolates were inhibited by SPR206 at MIC values of ≤2 mg/L. The *P. aeruginosa* MDR, XDR, and DTR subsets were completely inhibited by SPR206 (MIC_50/90_, 0.25/0.5 mg/L) at ≤1 mg/L (Table 1). SPR206 (MIC_50/90_, 0.25/0.25 mg/L) had MIC results fourfold lower than colistin (MIC_50/90_, 1/1 mg/L) when tested against all *P. aeruginosa* (Table 2 and Fig. 1). No *P. aeruginosa* isolates in this study were resistant to colistin (Table 2). Ceftazidime-avibactam (81.6–95.2% susceptible) and ceftolozane-tazobactam (90.2–96.9% susceptible) were the most active agents tested against the *P. aeruginosa* subset.

**TABLE 2:** Antimicrobial activity of SPR206 and comparator agents against gram-negative bacterial isolates by region^*g*^^*,^h^*^

Organism group/agent	Overall			Eastern Europe			Western Europe				USA		
	MIC_50/90_	% Susceptible		MIC_50/90_	% Susceptible		MIC_50/90_	% Susceptible			MIC_50/90_	% Susceptible	
		CLSI	EUCAST		CLSI	EUCAST		CLSI	EUCAST			CLSI	EUCAST
*P. aeruginosa*	*n* = 898			*n* = 133			*n* = 315				*n* = 450		
SPR206	0.25/0.25			0.25/0.25			0.25/0.25				0.25/0.25		
Colistin	1/1		100	1/1		100	1/1		100		1/1		100
Meropenem	0.5/8	78.3	78.3^*b*^	1/16	70.7	70.7^*b*^	0.5/8	80.0	80.0^*b*^		0.5/8	79.3	79.3^*b*^
Ceftazidime	2/32	79.5		2/>32	72.9		2/32	82.5			2/32	79.3	
CZA	2/8	95.2	95.2	2/8	92.5	92.5	2/4	96.8	96.8		2/8	94.9	94.9
PTZ	4/128	76.8		8/>128	71.4		4/128	81.6			4/128	75.1	
CXT	0.5/2	95.7	95.7	0.5/4	90.2	90.2	0.5/2	96.2	96.2		0.5/2	96.9	96.9
Amikacin	4/8	96.0^*c*^	96.0^*d*^	4/16	91.0^*c*^	91.0^*d*^	4/8	96.2^*c*^	96.2^*d*^		4/8	97.3^*c*^	97.3^*d*^
Tobramycin	0.5/1	90.4	93.4^*d*^	0.5/>16	86.5	88.7^*c*^	0.5/2	89.8	94.3^*d*^		0.5/1	92.0	94.2^*d*^
Levofloxacin	0.5/8	72.0		0.5/32	66.9		0.5/4	74.9			0.5/8	71.6	
*Acinetobacter* spp.^*a*^	*n* = 724			*n* = 183			*n* = 254				*n* = 287		
SPR206	0.12/0.5			0.12/16			0.12/0.5				0.12/0.5		
Colistin	0.5/2		92.5	0.5/>8		84.2	0.5/1		93.7		0.5/2		96.9
AMS	8/>64	54.0		64/>64	25.1		8/>64	51.2			4/32	74.9	
Meropenem	1/>32	53.9	53.9^*b*^	>32/>32	26.2	26.2^*b*^	1/>32	51.6	51.6^*b*^		0.5/>32	73.5	73.5^*b*^
Ceftazidime	16/>32	49.7		>32/>32	20.2		16/>32	46.9			8/>32	71.1	
PTZ	32/>128	48.6		>128/>128	21.5		128/>128	48.8			2/>128	65.7	
Amikacin	4/>32	62.4	59.8^*d*^	>32/>32	32.8	27.9^*d*^	4/>32	57.1	55.9^*d*^		4/>32	86.1	83.6^*d*^
Tobramycin	1/>16	60.8	60.8^*d*^	>16/>16	32.2	32.2^*d*^	1/>16	54.7	54.7^*d*^		1/>16	84.3	84.3^*d*^
Tigecycline	0.5/4			2/8			0.5/4				0.25/4		
Levofloxacin	0.5/>32	51.4	50.4	16/>32	22.4	21.9	1/>32	50.8	49.2		0.25/32	70.4	69.7
Enterobacterales^*f*^	*n* = 2,734			*n* = 364			*n* = 994				*n* = 1,366		
SPR206	0.06/0.25			0.06/1			0.06/0.25				0.06/0.25		
Colistin	0.25/0.25		95.2	0.25/2		90.9	0.25/0.25		96.9		0.25/0.25		95.2
Meropenem	0.03/0.06	96.6	97.0^*b*^	0.03/1	90.1	90.1^*b*^	0.03/0.06	98.0	98.1^*b*^		0.03/0.06	97.4	98.1^*b*^
Imipenem	≤0.12/0.5	96.4	97.0	≤0.12/1	90.1	90.6	≤0.12/0.5	97.4	98.1		≤0.12/0.5	97.4	97.9
Aztreonam	0.12/>16	75.4	73.8	0.25/>16	60.2	58.6	0.12/>16	75.7	74.0		0.12/>16	79.4	77.8
Ceftriaxone	0.12/>8	73.7	73.7^*b*^	0.25/>8	58.3	58.3^*b*^	≤0.06/>8	74.3	74.3^*b*^		≤0.06/>8	77.4	77.4^*b*^
Ceftazidime	0.25/>32	76.9	73.7	0.5/>32	60.7	58.8	0.25/>32	78.7	73.5		0.25/>32	80.1	78.0
CZA	0.12/0.5	99.5	99.5	0.12/1	97.9	97.9	0.12/0.5	99.8	99.8		0.12/0.5	99.6	99.6
PTZ	2/128	78.7	78.7	4/>128	67.4	67.4	2/128	79.5	79.5		2/64	81.3	81.3
CXT	0.25/4	88.4	88.4	0.25/>16	78.1	78.1	0.25/4	89.7	89.7		0.25/2	90.3	90.3
Amikacin	2/4	92.8	96.9^*d*^	2/8	83.7	90.1^*d*^	2/4	94.7	97.4^*d*^		2/4	94.0	98.5^*d*^
Tobramycin	0.5/8	87.1	87.1^*d*^	1/>16	73.0	73.0^*d*^	0.5/4	89.2	89.2^*d*^		0.5/4	89.3	89.3^*d*^
Tigecycline	0.25/0.5	98.4^*e*^		0.25/1	97.6^*e*^		0.25/0.5	98.8^*e*^			0.25/0.5	98.3^*e*^	
Levofloxacin	0.06/8	81.3	81.3	0.06/32	65.0	65.0	0.06/4	83.2	83.2		0.06/8	84.4	84.4
SXT	≤0.12/>4	78.2	78.2	≤0.12/>4	61.5	61.5	≤0.12/>4	79.7	79.7		≤0.12/>4	81.7	81.7
CRE	*n* = 91^*e*^			*n* = 37			*n* = 21				*n* = 33		
SPR206	0.06/32			0.5/64			0.06/0.5				0.06/0.5		
Colistin	0.25/>8		78.0	1/>8		59.5	0.25/0.5		90.5		0.25/0.5		90.9
Meropenem	32/>32	2.2	8.8^*b*^	>32/>32	0.0	0.0^*b*^	16/>32	9.5	9.5^*b*^		8/>32	0.0	18.2^*b*^
Imipenem	8/>8	2.2	6.6	8/>8	2.7	5.4	8/>8	0.0	9.5		8/>8	3.0	6.1
Aztreonam	>16/>16	5.5	3.3	>16/>16	2.7	2.7	>16/>16	14.3	9.5		>16/>16	3.0	0.0
Ceftriaxone	>8/>8	3.3	3.3^*b*^	>8/>8	0.0	0.0^*b*^	>8/>8	14.3	14.3^*b*^		>8/>8	0.0	0.0^*b*^
Ceftazidime	>32/>32	4.4	2.2	>32/>32	2.7	0.0	>32/>32	9.5	9.5		>32/>32	3.0	0.0
CZA	1/>32	83.5	83.5	2/>32	78.4	78.4	1/8	90.5	90.5		1/>32	84.8	84.8
PTZ	>128/>128	1.1	1.1	>128/>128	0.0	0.0	>128/>128	4.8	4.8		>128/>128	0.0	0.0
CXT	>16/>16	4.4	4.4	>16/>16	0.0	0.0	>16/>16	14.3	14.3		>16/>16	3.0	3.0
Amikacin	8/>32	42.9	53.8^*d*^	>32/>32	16.2	21.6^*d*^	4/32	61.9	66.7^*d*^		4/>32	60.6	81.8^*d*^
Tobramycin	>16/>16	18.7	18.7^*d*^	>16/>16	2.7	2.7^*d*^	16/>16	28.6	28.6^*d*^		16/>16	30.3	30.3^*d*^
Tigecycline	1/2	92.3^*e*^		1/2	94.6^*e*^		0.5/2	95.2^*e*^			1/4	87.9^*e*^	
Levofloxacin	32/>32	13.2	13.2	32/>32	0.0	0.0	32/>32	14.3	14.3		4/>32	27.3	27.3
SXT	>4/>4	18.7	18.7	>4/>4	2.7	2.7	>4/>4	9.5	9.5		>4/>4	42.4	42.4

^*a*^ Includes *A. baumannii-calcoaceticus* species complex (627), *A. beijerinckii* (1), *A. berezinae* (14), *A. courvalinii* (4), *A. gerneri* (1), *A. guillouiae* (1), *A. gyllenbergii* (1), *A. haemolyticus* (1), *A. johnsonii* (8), *A. junii* (10), *A. lwoffii* (7), *A. proteolyticus* (1), *A. radioresistens* (12), *A. schindleri* (1), *A. soli* (2), *A. ursingii* (24), *A. variabilis* (1), *A. vivianii* (1), and *Acinetobacter* spp. (7).

^*b*^ Using non-meningitis breakpoints.

^*c*^ Using UTI only breakpoints.

^*d*^ For infections originating from the urinary tract.

^*e*^ Susceptibility based on the US FDA breakpoint.

^*f*^ Excludes species intrinsically resistant to polymyxins (i.e., *Morganella* spp., *Providencia* spp., *Proteus* spp., and *Serratia* spp.) and includes *Citrobacter amalonaticus/farmeri* (1), *Citrobacter freundii* species complex (120), *C. koseri* (120), *Cronobacter sakazakii* (1), *Enterobacter cloacae* species complex (428), *Escherichia coli* (850), *Hafnia alvei* (3), *Klebsiella aerogenes* (130), *K. oxytoca* (220), *K. pneumoniae* (850), *K. variicola* (6), *Kluyvera ascorbata* (1), *Pantoea agglomerans* (1), *Pluralibacter gergoviae* (1), and *Raoultella ornithinolytica* (2).

^*g*^ Abbreviations: CAZ, ceftazidime-avibactam; PTZ, piperacillin-tazobactam; CXT, ceftolozane-tazobactam; AMS, ampicillin-sulbactam; SXT, trimethoprim-sulfamethoxazole; CRE, carbapenem-resistant Enterobacterales (includes one *Citrobacter freundii* species complex, eight *Enterobacter cloacae* species complex, one *Escherichia coli*, five *Klebsiella aerogenes*, three *K. oxytoca*, 71 *K. pneumoniae*, and two *Serratia marcescens*); Eastern Europe/Mediterranean region (Czech Republic, Greece, Hungary, Poland, Romania, and Slovenia), Turkey, and Israel; Western Europe region (Belgium, France, Germany, Ireland, Italy, Portugal, Spain, Sweden, Switzerland, and United Kingdom).

^*h*^ Empty cells indicate that the breakpoints were not available for interpretation.

### *Acinetobacter* spp.

SPR206 MIC_50_ of 0.12 mg/L and MIC_90_ of 0.5 mg/L were obtained against *Acinetobacter* spp., and 94.5% of these isolates were inhibited at ≤2 mg/L, and 90.4 and 87.9% of the MDR (MIC_50/90_, 0.12/2 mg/L) and XDR (MIC_50/90_, 0.12/8 mg/L) subsets were inhibited by SPR206 at MIC values of ≤2 mg/L (Table 1). Overall, SPR206 (MIC_50/90_, 0.12/0.5 mg/L) had MIC results fourfold lower than colistin (MIC_50/90_, 0.5/2 mg/L) and four to eightfold lower than tigecycline (MIC_50/90_, 0.5/4 mg/L) when tested against all *Acinetobacter* spp. (Table 2 and Fig. 1). Other comparator agents had MIC_90_ values of >16 mg/L (Table 2). Similar SPR206 MIC_50/90_ values of 0.12/0.5 mg/L were seen against the sets of isolates recovered from US and W-EUR institutions. However, SPR206 MIC_90_ result against isolates from E-EUR (MIC_50/90_, 0.12/16 mg/L) medical sites was 32-fold higher than isolates from the US and W-EUR. A total of 15.8 and 6.3% of isolates from E-EUR and W-EUR were colistin-nonsusceptible (Table 2).

### Enterobacterales

The SPR206 MIC_50_ and MIC_90_ values were 0.06 and >64 mg/L, respectively, when tested against all 3,228 Enterobacterales isolates. When Enterobacterales species that are intrinsically resistant to polymyxins were excluded (e.g., *Proteus* spp., *Morganella* spp., *Providencia* spp., and *Serratia* spp.), the SPR206 MIC_50_ and MIC_90_ values were 0.06 and 0.25 mg/L, respectively, and SPR206 inhibited 95.4% of the 2,734 isolates at MIC values of ≤2 mg/L (Table 1). The SPR206 MIC_50_ value (MIC_50/90_, 0.06/0.25 mg/L) against this subset was fourfold lower than colistin (MIC_50/90_, 0.25/0.25 mg/L) (Table 2 and Fig. 1). Other agents, such as meropenem (MIC_50/90_, 0.03/0.06 mg/L), imipenem (MIC_50/90_, ≤0.12/0.5 mg/L), ceftazidime-avibactam (MIC_50/90_, 0.12/0.5 mg/L), and tigecycline (MIC_50/90_, 0.25/0.5 mg/L) had low MIC_90_ values against this subset (Table 2).

Equivalent MIC_50_ (0.06 mg/L) values for SPR206 were obtained against CRE and MDR isolates, whereas MIC_90_ values were 32 and 64 mg/L, respectively (Table 1). Twenty CRE exhibited SPR206 MIC values ≥ 8 mg/L, which included 17 *K. pneumoniae* (8–>64 mg/L), two *S. marcescens* (>64 mg/L), and one *Enterobacter cloacae* (32 mg/L) (Table 1). MIC_50_ of 0.06 mg/L and MIC of_90_ 0.5 mg/L were obtained against CRE isolates from centers located in the US and Western Europe (W-EUR) regions (Table 2). The SPR206 MIC_50_ and MIC_90_ values of 0.5 and 64 mg/L were obtained against CRE isolates from sites located in the Eastern Europe/Mediterranean region (E-EUR), respectively. Similar regional variations in MIC results were observed for colistin when tested against CRE isolates, with MIC_50_ and MIC_90_ values of 0.25 and 0.5 mg/L, respectively, against isolates recovered from W-EUR and US centers. Elevated colistin MIC results (MIC_50/90_, 1/>8 mg/L) were noted against isolates from E-EUR (Table 2), as these regions, mostly Turkey (58.8%; 10/17), contributed 88.2% (15/17) of colistin-nonsusceptible isolates within both E-EUR and W-EUR (data not shown). Ceftazidime-avibactam (78.4–90.5% susceptible) and tigecycline (87.9–95.2% susceptible) were the only agents with significant activity against this CRE subset; some isolates were susceptible to colistin (59.5–90.9% susceptible) and amikacin (21.6–81.8% susceptible according to EUCAST breakpoints).

## DISCUSSION

This study quantified the *in vitro* activity of the novel polymyxin analog, SPR206, and broad-spectrum comparator agents against a large and comprehensive set of Enterobacterales, *P. aeruginosa*, and *Acinetobacter* spp. Surveillance isolates collected from multiple infection sources in patients in centers in the US, Europe, Israel, and Turkey during 2021. SPR206 (MIC_50/90_, 0.06/0.25 mg/L) showed potent *in vitro* activity against Enterobacterales, inhibiting 95.4% of these species at the colistin’s EUCAST susceptible breakpoint of ≤2 mg/L. A similar activity was also seen for SPR206 when tested against *P. aeruginosa* (MIC_50/90_, 0.25/0.25 mg/L) and *Acinetobacter* spp. (MIC_50/90_, 0.12/0.5 mg/L), inhibiting 94.5–99.8% of these organisms at MIC values of ≤2 mg/L.

SPR206 (MIC_50_, 0.06-0.25 mg/L) was also active against the three MDR subsets; however, SPR206 was less potent against the overall CRE and MDR subsets of Enterobacterales (MIC_90_, 32–64 mg/L), and MDR and XDR *Acinetobacter* spp. (MIC_90_, 2–8 mg/L). In general, the increased MIC_90_ values resulted from isolates originating from the E-EUR region, whereas the MIC_90_ values of 0.5 mg/L were obtained for SPR206 against the subsets from the US and W-EUR medical sites. This regional variation in activity was also observed with colistin and other comparators, with most agents showing less activity against surveillance isolates from E-EUR. This was likely due to the frequency of CRE, MDR, and XDR isolates in E-EUR, including colistin-nonsusceptible isolates compared to other regions. These findings correspond with other studies that showed higher and rising resistance rates in E-EUR (25–28).

Predictably, elevated SPR206 MIC results were observed against those Enterobacterales species intrinsically resistant to polymyxins, such as *Proteus* spp., *Morganella* spp., and *Providencia* spp. These findings are comparable to previous studies that found a similar SPR206 activity against contemporary collections of Enterobacterales, *P. aeruginosa*, and *Acinetobacter* spp. These findings demonstrate that, even with molecule structural changes that differentiate SPR206 from other polymyxins, cross-resistance between polymyxins and SPR206 and other novel polymyxins, such as QPX9003, may exist (15, 29).

Globally, the increase in antimicrobial agent resistance, including to last-resort options like carbapenems, and the prevalence of various resistance phenotypes in gram-negative organisms and isolates causing DTR infections underscore the need to develop potent and safer treatment options that are effective against resistant bacteria. Overall, this study demonstrated that SPR206 has potent activity against a large collection of Enterobacterales, *P. aeruginosa*, and *Acinetobacter* spp., including resistance subsets. SPR206 also exhibited similar or superior potency compared to colistin as well as agents from other antimicrobial classes tested in this study. Previous clinical studies demonstrated the safety of SPR206 over ascending doses in healthy volunteers (11, 30), of which results also reported a *Cmax* in plasma of 4.4 mg/L and, along with those results presented here, may anticipate a dosing regimen that may exceed requirements for clinical efficacy. These findings provide support for the clinical development of SPR206 as a potential option for treating infections caused by resistant gram-negative bacteria.

### Conclusion

This study generated *in vitro* MIC results for SPR206 and comparator agents against a broad collection of contemporary surveillance isolates and established a comprehensive benchmark for the activity and spectrum of SPR206. SPR206 demonstrated *in vitro* activity against Enterobacterales, *P. aeruginosa*, and *Acinetobacter* spp., including those with resistance phenotypes. These results, along with an enhanced safety profile, warrant the clinical development of SPR206 as an alternative for treating infections caused by MDR organisms and those causing DTR infections.
